# Importance of Sex Differences in Impulse Control and Addictions

**DOI:** 10.3389/fpsyt.2015.00024

**Published:** 2015-02-18

**Authors:** Marci R. Mitchell, Marc N. Potenza

**Affiliations:** ^1^Department of Psychiatry, Yale University School of Medicine, New Haven, CT, USA; ^2^Department of Neurobiology, Yale University School of Medicine, New Haven, CT, USA; ^3^Child Study Center, Yale University School of Medicine, New Haven, CT, USA; ^4^CASAColumbia, Yale University, New Haven, CT, USA

**Keywords:** sex differences, hormone effects, gender, impulsivity, addictions

Historically, sex- or gender-related differences in addictions have been understudied. When neglected, both sexes may not receive the full benefit of medical research. Although hormone fluctuations in women are rarely investigated with respect to treatments, levels of estrogen and progesterone may have large impacts on the efficacies of behavioral or pharmaceutical interventions (1–7). The National Institutes of Health (NIH) have been advocating for investigating gender-related differences and hormonal influences (8), including with respect to impulse control and its contributions to addictions. Despite the importance of studying sex differences, the standard integration of sex-difference considerations, including in preclinical research using cell lines and animals, has yet to occur.

Sex differences are present in personality traits and behaviors, such as impulsivity, that have been associated with addictions (both substance and non-substance). Impulsivity has been defined as a tendency to act with little foresight or little consideration of future consequences (9, 10). Impulsivity is a complex construct that may be separated into specific factors; two main domains that can be measured in the laboratory include impulsive action and impulsive choice (11). Both impulsive action and choice have been associated with drug use, in both a predictive fashion and as a result of drug use (12, 13). Work investigating sex differences in impulsive action in both animals and humans has shown mixed results (14). The mixed findings may in part relate to sex hormones, with females displaying fluctuating levels of impulsivity dependent on cycle phase and estrogen levels (14).

Impulsive choice has been measured in the laboratory using delay-discounting tasks (13, 15–17). While multiple studies suggest that men may be more impulsive than women, careful investigation of specific facets suggest otherwise. Women may display greater discounting rates than men (i.e., greater choice impulsivity); however, reward type is relevant as men have been found to discount real money more rapidly than women, with women discounting hypothetical rewards more rapidly than men (18). Among adolescents, female smokers appear more impulsive than male smokers, but male control subjects appear more impulsive than female control subjects (19). Consistent with findings from Kirby and Marakovic (18); Heyman and Gibb (20) found that female smokers also tend to discount the value of hypothetical rewards more rapidly than do males.

Among heavy drinkers, women exhibit poorer inhibitory control than men (21, 22). A study investigating the neural correlates of impulsivity in non-abusing individuals who were family-history positive for alcohol abuse found that those who are family-history positive show greater recruitment of brain regions involved in addiction, inhibitory control, and executive function compared to those without family histories of alcoholism; however, this effect was driven by males (23). Had gender differences not been built into the experimental design, such a finding would not have been identified. Although there exist strong associations between drug use and impulsivity in both humans and animals, with impulsivity increasing the propensity for drug use and vice-versa (12, 13, 24, 25), few studies have investigated sex differences, particularly in preclinical work. The possible roles for cycle phase or circulating hormones in delay-discounting-task performance warrant further study.

Impulsivity and behavioral performance in impulsivity tasks does not always differ between men and women; however, that does not mean that both sexes are achieving similar performance in the same way. Even when men and women perform comparably in inhibitory tasks, different neurobiologies may underlie the behaviors. For example, in a recent study of gender-related differences in neural factors associated with performance of the stop-signal task, men tended to show more activation in the lentiform nucleus, parahippocampal gyrus, posterior and anterior cingulate cortices, middle and medial frontal cortices, and thalamus, compared to women, despite similar performance on the task (26). In general, men and women display different brain connectivity patterns, both in adolescence and adulthood. One study found that men show greater within-hemispheric connectivity and women show greater across-hemispheric activity, suggesting that male brains may be better suited to facilitate connectivity between perception and coordinated action, whereas female brains may be better suited to facilitate communication between analytical and intuitive processes (27). As neurobiological differences in males and females start in early stages of development (28, 29), it may be difficult to determine which differences are the result of genetics, which are influenced by cycling hormones and which may arise through interactions and other processes. Some differences may arise from how similarly conserved genes across the sexes are translated and expressed differently depending on sex (30).

Although men and women may use the same drugs and display the same behavioral addictions, frequencies may vary by drugs and behaviors (31). Furthermore, addictions may present differently, have different courses and patterns of comorbidity, be driven by different motivations, and have different factors leading to relapse (14, 32, 33). Males typically have higher rates of drug use and are more likely to develop dependence or abuse; however, women may have transition from initial use to dependence more quickly. Preclinical and clinical data suggest an enhanced vulnerability to drug use with greater acquisition of drug self-administration in females as compared to males (32, 34–40).

Given apparent sex differences in susceptibilities to drug use, sex differences in the approach to treating drug use and drug-use disorders are important to consider in order to optimize interventions for each sex. A recent study investigating sex differences in the efficacy of disulfiram in cocaine and alcohol dependence found that women, compared to men, had poorer treatment outcomes on several measures of cocaine use during treatment and at post-treatment follow-up, which was primarily accounted for by disulfiram being less effective in women than men (41). Gonadal hormones may influence relationships with treatment outcomes as estrogen may enhance the rewarding properties of drugs whereas progesterone may be more protective and attenuate drug-rewarding effects (5, 7, 42). Severity of withdrawal symptoms may vary based on menstrual-cycle phase (1–4). Moreover, estrogen interactions with dopamine transmission or the hypothalamic-pituitary-adrenal (HPA) axis may partially underlie facilitative effects (43–45). For example, females may be more affected by stress and report stress as a reason for drug use and relapse; therefore, greater activation of the HPA axis through stress has the potential to interact with circulating estrogen and monoamine neurotransmitters such as dopamine and serotonin (both implicated in rewarding and motivational aspects of drug use and impulse control) (34, 46, 47).

Women may be more likely to engage in addictive behaviors for negative reinforcement reasons (e.g., escape from stress) whereas men may be more likely to engage in addictive behaviors for positive reinforcement reasons (e.g., seeking a high), and these motivational differences may reflect different biologies and/or result in different clinical presentations. For example, studies link differences in cortico-striato-limbic activations in cocaine dependence to stress cues in women and drug cues in men (48). Additionally, women more frequently than men present with other mental health issues, such as trauma and depression, that co-occur with addictions (33).

Sex differences may extend to non-substance or behavioral addictions like gambling disorder. Women with gambling problems often display a “telescoping” effect similar to women with substance addictions, whereby females often initiate recreational behavior at a later age than men but progress more quickly into problematic gambling (49, 50). Males tend to develop problems with “face-to-face” forms of gambling (e.g., poker or blackjack), whereas females are more likely to develop problems with less personally interactive forms [e.g., bingo, keno, electronic-gambling (slot) machines], with differences appearing to relate to impaired control over gender-related behavioral preferences evident in recreational gamblers (49, 51). Taken together, data suggest important gender-related differences exist that warrant consideration in optimizing policy, prevention, and treatment initiatives.

When investigating the relationships between sex, hormones, impulsivity, and addictions, it will be important to consider research designs. Impulsivity is a multi-faceted construct and therefore using tasks assessing specific aspects of impulsivity is important (52, 53). Additionally, the reinforcer presented may also be an important variable as males and females may have different motivations relating to consumption of specific reinforcers, and women may discount more quickly than men when rewards are hypothetical. In rodents, no differences were found between males and females in premature responding when the reward was food; however, when the reward was cocaine, female rats made significantly more premature responses (54). It is unclear whether the increase in premature responding in female rats for cocaine was related to cycle phase. High estrogen levels may attenuate impulsive action and depleting male rats of testosterone may decrease impulsive action (55), but high estrogen levels have also been associated with increased sensitivity to cocaine (56). Therefore, it is important to determine whether there are fluctuating levels of impulsivity across different cycle phases and how these might relate to addictions. Additionally, understanding and studying genetic differences (and similarities) between men and women that may underlie behavior and neural activity is important. One way to disentangle potential roles of hormones and genetic factors relating to impulsivity and addiction involves manipulating hormone levels by administering or blocking cycling hormones and using hormone replacement therapy, particularly in females who are in menopause. Moreover, in preclinical models, the use of ovariectomized animals and controlled administration or release of various hormones is possible. Preclinical studies may effectively disentangle influences of genotype (XX, XY) from gonadal phenotype (ovaries, testes) with respect to impulsivity-related and addiction-related behaviors (57). Applying these techniques in longitudinal studies across the life-span in both females and males could provide important insight into developmental sex differences in impulsivity and addiction, and these findings may inform human research and efforts to develop more effective policy, prevention, and treatment interventions.

In summary, data demonstrate the importance of studying sex differences in addictions and impulsivity and their interactions. While research has progressed in these areas, there remains a deficit in understanding sex differences. While NIH has promoted the study of males and females in clinical populations, sex differences are not uniformly and systematically investigated and influences of circulating hormones are not routinely documented. Therefore, it is difficult to determine which differences may link to hormones and which may link to genetic differences between the sexes. In preclinical research, sex differences are often neglected, which may limit the translation of preclinical findings into clinical settings. Routine considerations of sex differences in preclinical and clinical research settings will help advance translational efforts and improve prevention, treatment, and policy initiatives.

## Conflict of Interest Statement

The authors declare that the research was conducted in the absence of any commercial or financial relationships that could be construed as a potential conflict of interest.
